# Effect of maternal calcium intake during pregnancy on children's blood pressure: A systematic review of the literature

**DOI:** 10.1186/1471-2431-7-15

**Published:** 2007-03-26

**Authors:** Eduardo Bergel, Aluisio JD Barros

**Affiliations:** 1 Department of Mother & Child Health Research, Institute for Clinical Effectiveness and Health Policy, School of Public Health, School of Medicine, University of Buenos Aires, Marcelo T de Alvear 2202, (C1122AAJ) Buenos Aires, Argentina; 2 Department of Social Medicine, Federal University of Pelotas, C.P. 464 – 96001-970, Pelotas, Brazil

## Abstract

**Background:**

Calcium supplementation during pregnancy has been shown to reduce the incidence of hypertension in the mother, but the effects on the offspring are uncertain. Assessing the impact on the offspring is very important given the now large body of evidence indicating that blood pressure levels in childhood and young adulthood can be influenced by factors operating during fetal life. We conducted a systematic review of the literature to summarize the evidence supporting an association between maternal dietary calcium intake during pregnancy and blood pressure in the offspring.

**Methods:**

A systematic review was performed to identify randomized, quasi-randomized and cohort studies reporting the relationship between offspring blood pressure or incidence of hypertension and levels of maternal dietary calcium intake during pregnancy, either from supplements (i.e. pills) or food. MEDLINE, EMBASE and the Cochrane Library Registry were searched for relevant trials.

**Results:**

Two randomized trial and three observational studies were identified and included in this review. In 4 of the 5 studies, loss to follow-up was a serious concern. There was heterogeneity between the studies, particularly those conducted on children below 12 month of age. Results were more consistent among the studies including older children (1 to 9 years) where a higher maternal calcium intake was associated with a reduction of -1.92 mm Hg (95% CI -3.14 to -0.71) in offspring systolic blood pressure. One large randomized trial found a clinically and statistically significant reduction in the incidence of hypertension in 7-year-old children (RR = 0.59, 95% CI 0.39 to 0.90).

**Conclusion:**

There is evidence in the literature to support an association between maternal calcium intake during pregnancy and offspring blood pressure. However, more research is needed to confirm these finding given the small sample sizes and the methodological problems in many of the studies conducted so far. More studies on populations with calcium deficit are also needed. If confirmed, these findings could have important public health implications. Calcium supplementation during pregnancy is simple and inexpensive and may be a way to reduce the risk of hypertension and its sequels in the next generation.

## Background

Increased dietary calcium intake has been associated with lower blood pressure among children, adults and pregnant women [1,2]. The effect seems to be more evident among individuals with low calcium intake [3-6]. Some recent experimental and observational studies in humans and animals have reported an association between maternal calcium intake during pregnancy and blood pressure in the offspring [5,6], but others have not [1,7]. These findings follow a large body of evidence indicating that blood pressure levels in childhood and young adulthood are influenced by factors operating early in life [7-10] and are associated with later cardiovascular disease and mortality [11]. We conducted a systematic review and meta-analysis of studies reporting blood pressure levels in offspring of mothers who were either enrolled in a trial of calcium supplementation during pregnancy or included in a study on maternal calcium intake during the index pregnancy.

## Methods

### Types of studies

This review includes randomized and quasi-randomized controlled trials. We pre-specified that if the evidence from randomized trials was insufficient (i.e. small trials, trials of bad quality, etc.) to assess the effect of the intervention, then data from observational studies (e.g. cohort studies) would be considered for inclusion. Studies with historical controls and ecological studies were excluded, as the data provided by these are unreliable for determining causation and/or association.

Studies should provide an estimate of the incidence of hypertension, or the mean difference in offspring blood pressure between levels of maternal calcium dietary intake during pregnancy, or should enable this information to be computed from data extracted from the article.

### Types of participants

Offspring of mothers included in studies assessing the association between calcium intake during pregnancy and offspring blood pressure.

### Types of intervention or exposure

Maternal dietary calcium intake during pregnancy, from supplements (i.e. pills) or food.

### Types of outcome measures

Offspring diastolic and systolic blood pressure in mm Hg; incidence of hypertension.

### Search strategy for identification of studies

1. MEDLINE and EMBASE (1966 to 2005) were searched in December 2005 using the following search strategy

#1 Search calcium [Title] 84110

#2 Search pregnan* 568541

#3 Search "blood pressure" 247471

#4 Search hyperten* 241702

#5 Search #1 AND #2 AND (#3 OR #4) 253

2. All databases included in The Cochrane Library (issue 4, 2005) were searched with a search strategy equivalent to the Medline strategy.

3. Reference lists of all the studies that went into the pool of retrieved studies, including those of other reviews, were examined to identify any further studies. We did not implement a standardized strategy to find unpublished studies.

### Selection of studies and data extraction

The titles, abstracts and descriptor terms of all material downloaded from the electronic searches were read and irrelevant reports were discarded. All citations identified were then inspected to establish the relevance of the article according to the inclusion criteria. Where there was uncertainty about relevance, the full article was obtained. Studies were reviewed for relevance on the basis of study design, types of participants, exposures and outcome measures. Standardized data extraction forms were used, one for clinical trials and one for cohort/cross-sectional studies. The following characteristics were extracted from each study included:

a) Administrative details: identification; author(s); published or unpublished; year of publication; year in which study was conducted; details of other relevant papers cited.

b) Details of study: study design; method(s) of recruitment; inclusion and exclusion criteria; number of participants assessed for eligibility, number excluded, number enrolled, number analyzed; type, duration, frequency and completeness of follow-up in the case of cohort studies; country and location of the study.

c) Characteristics of participants: age; location; details of intervention.

d) Crude and adjusted measures of effect, confidence intervals and p-values were extracted. When an adjusted analysis was performed, type of analysis and the list of covariates adjusted for were recorded.

e) The amount of maternal calcium intake was extracted and reported as the amount of elemental calcium in mg.

### Details of analysis

One study [12] did not report regression coefficients, but reported the correlation coefficient between maternal calcium intake and offspring blood pressure. Standard errors for the correlation coefficients were computed using the Fisher r-to-z transformation [13]. The correlation coefficients were transformed to standardized mean differences and multiplied by blood pressure standard deviation to estimate the effect [14].

The meta-analysis was conducted using the Revman computer software package [15]. The amount of heterogeneity between studies was assessed by a formal statistical test (chi-square test), and by I2 [16]. The chi-squared test assesses whether observed differences between results are compatible with chance alone [17]. A low p-value provides evidence of heterogeneity of treatment effects [17]. The results of the chi-squared test might be misleading because it has low power for trials with small sample size, and I2 has been proposed as an additional tool for assessing heterogeneity [16]. I2 describes the percentage variability in effect estimates that is due to heterogeneity rather than sampling error (chance) and is not affected by the number of trials in the meta-analysis [17]. A value greater than 50% may be considered substantial heterogeneity. Mathematically, it is defined as I2 = [(Q - df)/Q] × 100%, where Q is the chi-squared statistic and df is the number of degrees of freedom [18,19].

### Quality assessment

Quality of randomized controlled trials was assessed following the method described in the Cochrane Collaboration Handbook [17]. For observational studies, quality was assessed following the recommendations of the Meta-analysis of Observational Studies in Epidemiology (MOOSE) guidelines [20].

## Results

Two randomized trials [21,22] and three observational studies [12,23,24] were included in the review. Three studies included infants less than one year of age [12,22,23] and four included children between 1 year and 9 years of age [12,21,22,24]. Table 1 describes the characteristics of the two randomized trials. Both studies were long-term follow-ups of randomized trials of calcium supplementation during pregnancy to prevent pre-eclampsia. In both studies the randomization procedure was adequate, subject and health professionals were blinded regarding calcium or placebo status, and baseline characteristics were similar between study arms. Belizan et al. [25] randomized 1194 women during early pregnancy to either 2000 grams of oral calcium supplementation or placebo. This study included only healthy primiparous women. Compliance with calcium supplementation was acceptable (more than 80%), and increased calcium intake in the intervention group was confirmed by measurements of urinary calcium excretion. A follow-up of this experimental cohort was conducted 7 years after the original trial [21]. The original study was conducted in three hospitals, two public and one private, but the follow-up only included the 614 participants from the private hospital (approximately 50% of the original sample). Randomization was stratified by center, and the baseline characteristics of those included in the follow-up were comparable between the trial arms. However, women excluded from the follow-up were younger and had lower socioeconomic status than those included. Loss to follow-up (16%) was acceptable for a long-term follow-up. This study used cut-off points for systolic and diastolic pressure, specific for sex, age and height, that corresponded to the 95th centiles given in tables developed by the US National Institutes of Health.

Hatton et al. [22] randomized 4,589 women during early pregnancy to either 2000 grams of oral calcium supplementation or placebo. A detailed clinical and laboratory evaluation was conducted before trial entry, and women with complications during pregnancy or signs or history of calcium metabolism disorders were excluded. Furthermore, a baseline compliance test was used to exclude women with low compliance before trial entry. Two follow-up studies were conducted at 3 and 24 months post-partum. Patients from only one out of five medical centers were included in the follow-up (559 out 4589 subjects). Randomization was stratified by center, but no data are available on the baseline characteristics of the follow-up sample. Loss to follow-up was 53% at 3 months and 90% at 2 years. The authors acknowledge this to be a problem, adding that that a large proportion of the cohort had not reached two years of age by the end of the study. This is the main methodological limitation of this study. Sample size is also an issue, given the small number remaining for analysis. Calcium intake from other sources (i.e. prenatal supplements, diet) in the population from Belizan et al. [25] was estimated at 600 mg per day, well below the recommended level during pregnancy. In contrast, the reported calcium intake for the population in Hatton et al. [22] was over 1200 mg per day, within recommended levels. In summary, the two randomized trials included [21,22] were similar in respect of the characteristics of the intervention, study design and inclusion criteria, but differed in an important population characteristic (baseline calcium intake); also, loss to follow-up was a problem in one study [22].

Table 2 describes the characteristics of the three observational studies included in the review [12,23,24]. McGarvey et al. [12] designed their study to explore the association between infant blood pressure and maternal dietary intake of calcium, potassium and magnesium. Data on maternal prenatal diet were obtained by conducting a post-partum 116-item semi-quantitative food frequency questionnaire (FFQ). The authors measured the offspring blood pressure in hospital when the babies were 2–4 days old, and at home at 1, 6 and 12 months.

Gillman et al. [23] used the data from a cohort study of pregnant women conducted in the US. This study was designed to assess the effects of mother's diet on mother's and offspring's health. It assessed maternal calcium intake during the first and second trimesters using a validated semi-quantitative FFQ, and measured offspring blood pressure at birth and at 6 months. The authors reported figures for calcium from food sources and from prenatal supplements, and then performed two independent analyses accordingly. This seems to have been a non-pre-specified analysis. The strength of the evidence for this analysis is not clear because it was not stated whether this analysis strategy was pre-specified [26].

Morley et al. [24] used the data from a population-based survey in Tasmania designed to investigate sudden infant death syndrome. Mothers of all live-born twins during the study period were approached after birth for data collection, including nutritional supplement consumption during pregnancy. Data on calcium consumption from other sources (i.e. foods) were not available. Children were assessed at a mean age of 9 years and blood pressure was measured.

All three studies were conducted in developed countries, and average calcium intake was higher than the recommended calcium intake during pregnancy for the two studies with quantitative estimates [12,23].

It is not clear whether outcome assessment in any of the three observational studies was blinded to levels of exposure. Loss to follow-up was large (see table 2 for details). One of the studies [12] conducted follow-ups at 4 time points, but the sample size decreased for older infants. Less than 40% of the sample was available in the last follow-up at 1 year of age. The authors reported that one of the primary reasons for the decrease in sample size with age was that not all infants had reached 1 year by the end of the study.

All three observational studies attempted to adjust for confounding variables, but they differed markedly in the set of variables included. Two of them adjusted for blood pressure measurement conditions [12,23]. All three adjusted for socioeconomic factors (e.g. maternal education) and child age at assessment. One [12] reported estimates from more than one model, including one adjusted for dietary potassium and magnesium. Crude and adjusted estimates were similar in all three studies, suggesting that confounding was not a problem in exploring this association.

A funnel plot was produced to assess publication bias [27]. Because of the small number of studies, the figure was not very informative and was not included in this report. Publication bias cannot be excluded.

In summary, all five studies seem to have been well conducted. The main limitation in all the observational studies was loss to follow-up. For two of these studies, small sample size was also a problem. In one study, the focus of the analysis was a sub-group analysis.

**Figure 1:**
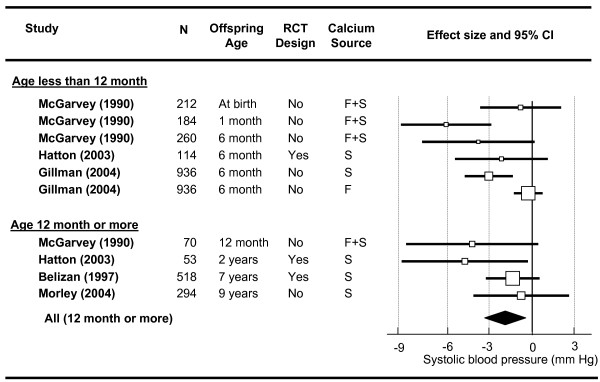
Maternal calcium supplementation during pregnancy and offspring systolic blood pressure, by offspring age at follow-up. Key: Calcium source: F from food, S from supplements. RCT, Randomized Controlled Trial. Lines are 95% confidence intervals. Boxes are proportional to the sample size of the trial.

Higher maternal calcium intake during pregnancy was associated with lower offspring systolic blood pressure in all studies, but the effect was statistically significant in only 3 of them [12,22,23] (see figure 1). There was heterogeneity between studies (I2 > 50%); this was large for studies conducted on children below 12 months of age (I2 = 53%), and small for studies on older children (I2 < 10%). Because of this heterogeneity, the analysis was stratified according to age, and a summary measure (meta-analysis) was obtained only for studies that reported on children aged 1 year or more. The results of this analysis are compatible with a reduction in SBP in the calcium group of -1.92 mm Hg (95% CI -3.14 to -0.71) (see figure 1).

In the largest randomized trial [21] there was a modest, statistically insignificant effect on systolic blood pressure, but a clinically and statistically significant effect on the incidence of high systolic blood pressure at 7 years of age (see table 3). This study also reported that the effect is stronger among overweight children; this was not observed by others, though the sample sizes were too small to exclude such a difference.

For children under 12 months, the data are less consistent (see table 4). In the same cohort, one study [12] found no effect at birth, a strong effect at 1 month and a moderate effect at 6 months. Another [23] reported that no effect was associated with calcium from foods, but a strong effect was found in association with (prenatal) calcium supplements. This analysis strategy did not seem to be pre-specified, and the authors failed to provide a convincing hypothesis to account for the finding. Finally, the follow-up at 3 months conducted by Hatton et al. [22] was too small to draw any conclusion.

## Discussion

This systematic review of the literature identified two randomized trials and three observational studies. The evidence provided by this body of research suggests an association between dietary calcium intake during pregnancy and offspring blood pressure. A good quality randomized trial found a large reduction in the incidence of hypertension in children at 7 years of age [21]. However, the same trial found a smaller effect on blood pressure as a continuous variable. A possible explanation for these findings is that the intervention produced a change in the shape of the blood pressure distribution, as opposed to a shift in mean blood pressure.

A meta-analysis that combined four studies on children over one year of age found a reduction in mean systolic blood pressure. This finding should be viewed with caution, since evidence obtained by combining small studies has previously been shown to be unreliable because of publication bias [28]. For infants under one year of age the evidence is contradictory and difficult to summarize.

Among the 5 studies reported, only two were randomized trials. The validity of the evidence from observational studies for assessing the effect of interventions is controversial [17]. The two randomized trials included in the review were multi-center trials and the randomization was stratified by center. The authors chose to follow up subjects from only one center. It can be assumed that because the randomization was stratified by center, the effect estimate will not be biased. However, an impact on external validity can be expected. For example, in one study, participants from the selected hospital were of higher socioeconomic status than those from the centers not included in the follow-up [21]. Apart for the methodological problems of the original articles, other limitations of this analysis should be pointed out. Four out of the five studies included in the review were conducted in developed countries, and on populations in which maternal calcium intake was adequate or even higher than the recommended levels during pregnancy. This is clearly not the ideal target for a nutritional intervention. Given the evidence that the effect of calcium might be apparent only when there is a deficit, the external validity of these results might be compromised [5].

The heterogeneity between studies also creates difficulties for interpreting the results. The sources and dose of dietary calcium vary widely among the observational studies, and so do the methods used to assess the amount consumed. There are also large differences between studies in infants' ages at assessment. It is well known that the determinant of blood pressure varies with age, and it has been shown that the impact of factors affecting the fetal environment are seen particularly after adolescence [29]. This problem is magnified because of the difficulties in measuring blood pressure accurately at early ages [30].

## Conclusion

In summary, there is evidence in the literature to support an association between maternal calcium intake during pregnancy and offspring blood pressure. However, more research is needed to confirm these findings, given the small sample sizes and the methodological problems of many of the studies conducted so far. New evidence should be derived from the long-term follow-up of large and well-conducted randomized trials of calcium supplementation during pregnancy. More studies on populations with calcium intake deficit are also needed. Assessing the effect of the intervention on other cardiovascular risk factors would also be an asset for future research. Calcium supplementation during pregnancy is simple and inexpensive, and if these findings are confirmed it could be a way to prevent hypertension and its sequels in the next generation.

## Competing interests

One of the authors (EB) authored one of the reports included in the systematic review [21].

## Authors' contributions

EB was the principal author responsible for the conception and design of the systematic review, screening of citations, data auditing, analysis and interpretation, and drafting and editing of the manuscript. AB drafted and edited the manuscript and made major contributions to the interpretation of the evidence and manuscript revisions. Both authors read and approved the final manuscript.

## Pre-publication history

The pre-publication history for this paper can be accessed here:



## Figures and Tables

**Table 1:** Characteristics of randomized controlled trials included in the review.

	**Belizan, 1997**	**Hatton, 2003**
**Methods**		
**Randomization**	Numbered, sealed opaque envelopes, containing randomization codes.	Numbered treatment packs in computer-generated simple randomization sequence.
**Lost to follow-up – women**	Of 593 (calcium) vs. 601 (placebo) enrolled, 14 vs. 13 were lost before starting treatment and excluded from analysis; 577 vs. 588 had at least partial follow up. Follow up was incomplete for 52 vs. 46, but delivery data were available in 17 vs. 12 of these, giving delivery data for 544 vs. 554.	calcium 132/2,295 vs. placebo 121/2,294.
**Lost to follow-up – offspring**	Of 614 randomized in one center (calcium 309/placebo 305), 301/299 completed the first study, 2/6 infant deaths and 1/0 maternal deaths had occurred, leaving 298/293 eligible for follow up. 289/285 were contacted, 10/5 refused to participate, 22/19 lived outside the country, and 257/261 were assessed.	559 randomized in study site. Of 497 invited to participate in the follow-up study, BP was measured in 260 infants at 3 months of age and 57 toddlers at 2 years of age.
**Participants**		
**Mothers**	Nulliparous women, < 20 weeks pregnant; blood pressure < 140/90 mmHg (mean of 5 measurements); no present or past disease; not taking medication; normal oral glucose tolerance tests.	Pregnant nulliparas (45% black, 35% non-Hispanic white, 17% Hispanic white). Passed compliance test (took 75% of placebo over 6–14 days); BP 134/84 mmHg or less; urine protein dipstick negative or trace; 13–21 weeks pregnant.
		Exclusion criteria: taking medications; obstetric or pre-existing diseases or personal characteristics which could influence study end-points, absorption or metabolism of calcium; any risk associated with calcium supplementation, or compliance; elevated serum creatinine (1.0 mg per decilitre or more) or calcium (10.6 mg per decilitre or more); renal disease; haematuria; history or family history of urolithiasis; frequent use of calcium supplements or antacids.
		Of 11,959 women screened, 5,703 excluded initially and a further 1,667 after the compliance test. The remaining 4,589 women were enrolled.
**Offspring**	Two public and one private hospital participated in the original trial, but only babies born in the private hospital were included in the follow-up study at 7 years of age.	Five participating medical centers participated in the original trial, but only babies born in one center (Oregon) were included in the follow-up study. Additional criteria for inclusion: mothers who completed the original trial, delivered an infant without serious problems, and who read english. Follow-up was completed at 12 week postpartum and at 2 years of age.
**Interventions**	2 g calcium as 500 mg calcium carbonate tablets, vs. identical looking placebo tablets. Compliance was 84% (calcium) and 86% (placebo).	2 g/day elemental calcium as calcium carbonate, or placebo. Taken until delivery, development of pre-eclampsia or suspicion of urolithiasis. All women took 50 mg calcium per day as normal supplementation and were asked to drink 6 glasses of water per day.
		Compliance was 64% in the calcium group and 67% in the placebo group. 20% of women took > 90% of the allocated treatment.
**Outcomes**	Systolic blood pressure, diastolic blood pressure, kidney stones, gall stones, hospital admissions	Systolic blood pressure, left ventricular wall mass. Diastolic blood pressure was measured at 2 years but group means were not reported.
**Allocation Concealment**	A	A

**Table 2:** Characteristics of observational studies included in the review.

	**McGarvey, 1990**	**Gillman, 2004**	**Morley, 2004**
**Country, region**	Rhode Island, USA	Massachusetts, USA	Tasmania, Australia
**Age at Follow-up**	At birth – 1 month – 6 month – 12 month	6 month	9 years
**Sample size**	212 – 184 – 114 – 70	936	294
**Outcome measures**	Systolic and diastolic blood pressure	Systolic blood pressure	Systolic and diastolic blood pressure, insulin, fasting glucose, triacylglicerol, cholesterol (total, HDL and LDL)
**Average maternal calcium intake during pregnancy (mg/day)**	1712	Total:1494 first trimester (FT), 1330 second trimester (ST). From prenatal supplements: 264 (FT), 203 (ST). From food: 1230 (FT), 1128 (ST).	NA
**Exposure measurement**	Maternal prenatal diet assessed postpartum in hospital by a 116 item semi quantitative food frequency questionnaire.	Maternal diet assessment with a semi quantitative food frequency questionnaire validated in pregnancy during the first trimester and second trimester. Analysis stratified by calcium from food and calcium from prenatal supplements.	Mothers of all live born twins in Tasmania were approached soon after birth. They were asked whether they had taken any nutritional supplements during pregnancy.
**Exposure categories, maternal calcium intake in mg**	Quartiles (1380, 1722, 2048) and correlation coefficients	Quartiles (Q4 - Q1 = 424 mg) and linear regression	Maternal calcium supplements during pregnancy (yes/no). Calcium content of supplements not stated.
**Blinding of exposure status and ascertainment of outcome.**	Unknown	Unknown	Unknown
**Lost to follow-up or invalid exclusions**	Total eligible population not stated. 212 mothers with prenatal dietary data included. Lost to follow-up by age at follow-up (%): 0 13 46 67	2128 total population. 462 missing maternal diet assessment, 232 did not consent, 381 did not attend follow-up visits, 77 outcome missing, 39 missing covariates.	Total eligible population not stated. 463 twin children recruited. 11had co-twin not available for recruitment. 23 pairs left Tasmania. 14 pairs could not be traced and 42 declined to participate.
**Adjustment**	Cuff size, observer, sleep/activity status. Age in days (At birth), body weight (at 6 and 12 month). Maternal race, parents education and occupation. Dietary potassium and magnesium	Energy intake, BP measurement conditions (cuff size, infant position, appendage used, machine model, infant state, clinic site)	Maternal age and education, twin pair birth order (in family), child age at assessment and sex. Study in twins.
		Maternal race, education, number of previous pregnancies, marital status, pregnancy body mass index and third trimester systolic blood pressure. Infant age and sex.	
**Comments**		Study reported two sets of results, for calcium from food and from prenatal supplements.	Study in twins.

**Table 3:** Maternal calcium intake during pregnancy and blood pressure in the offspring. Summary of results from the long term follow-up of randomized controlled trials.

**Outcome measure**		**Age at follow-up**	**calcium**		**placebo**		**Mean Difference (95%CI)**	**p value**
			**N**	**mean**	**N**	**mean**		
**Systolic Blood Pressure (mm Hg)**								
	**Belizan 1997**	7 years	257	103.9 (10.6)	261	105.3 (11.0)	-1.4 (-3.2 to 0.5)	0.14
	**Hatton 2003**	2 years	35	95.4 (7.6)	18	100.2 (7.9)	-4.8 (-9.2 to -0.3)	0.036
	**Hatton 2003**	3 month	130	111.4 (14.3)	130	113.6 (12.6)	-2.2 (-5.5 to 1.1)	0.20
**Diastolic Blood Pressure (mm Hg)**								
	**Belizan 1997**	7 years	257	65.4 (9.3)	261	65.8 (9.3)	-0.4 (-2.0 to 1.2)	0.63

			**n/N**	**(%)**	**n/N**	**(%)**	**Relative Risk (95%CI)**	

**High Blood Pressure – systolic**								
	**Belizan 1997**	7 years	29/257	(11.4)	50/261	(19.3)	0.59 (0.39 to 0.90)	0.01
**High Blood Pressure – diastolic**								
	**Belizan 1997**	7 years	26/257	(10.2)	33/261	(12.7)	0.80 (0.49 to 1.30)	0.41

**Table 4:** Maternal calcium intake during pregnancy and blood pressure in the offspring. Summary of results from observational studies.

**Outcome measure**	**Age at follow-up**	**Calcium Source†**	**Maternal calcium intake (mg)**	**N**	**Offspring blood pressure in mm Hg, by quartiles of maternal calcium intake**				**Crude effect size (95%CI)‡**	**p value**	Adjusted effect size (95% CI)*	**p value**
									
					**low**	**med-low**	**med-high**	**high**				
**Systolic blood pressure (mm Hg)**												
**McGarvey 1990**	At Birth	F+S	1712	212	71.5	70.8	70.2	69.9	-0.84 (-3.69 to 2.01)	0.56	NA	
**McGarvey 1990**	1 month	F+S	1712	184	82.4	81.8	77.9	75.5	-6.13 (-9.32 to -2.94)	< 0.01	-4.28 (-7.12 to -1.44)	< 0.01
**McGarvey 1990**	6 month	F+S	1712	114	87.0	85.7	83.5	84.1	-3.84 (-7.81 to 0.13)	0.06	-3.08 (-6.66 to 0.50)	0.08
	6 month	F			91.5	90.2	90.4	88.4	-0.30 (-1.29 to 0.69)	0.55	-0.04 (-1.10 to 1.00)	0.55
**Gillman 2004**	6 month	S	1494	936	91.1	90.3	91	90.3	-3.10 (-4.78 to -1.42)	< 0.01	-3.00 (-4.90 to -1.10)	< 0.01
**McGarvey 1990**	12 month	F+S	1712	70	89.6	85.8	85.3	86.2	-4.28 (-8.94 to 0.38)	0.72	-3.40 (-8.40 to 1.69)	0.14
**Morley 2004**	9 years	S	NA	294	96.4		95.6		-0.80 (-4.17 to 2.57)	0.64	-0.70 (-4.20 to 2.70)	0.70
**Diastolic blood pressure (mm Hg)**												
**McGarvey 1990**	At Birth	F+S	1712	212	42.1	42.6	41.8	41.7	0.21 (-2.64 to 3.06)	0.88	NA	
**McGarvey 1990**	1 month	F+S	1712	184	41.0	43.1	41.2	40.9	-0.95 (-3.72 to 1.82)	0.50	-1.91 (-4.96 to 0.87)	0.18
**McGarvey 1990**	6 month	F+S	1712	114	56.9	51.9	48.6	50.3	-5.89 (-9.95 to -1.83)	< 0.01	-4.28 (-7.90 to -0.66)	0.01
**McGarvey 1990**	12 month	F+S	1712	70	53.1	49.1	47.6	49.7	-4.80 (-9.39 to -0.01)	0.05	-5.19 (-10.37 to 0.01)	0.07
**Morley 2004**	9 years	S	NA	294	52.6		51.8		-0.80 (-2.80 to 1.20)	0.40	-0.90 (-2.90 to 1.10)	0.40

‡ Regression coefficient between maternal calcium intake and blood pressure.

* See table 2 for the list of variables of variables included in the models for each study.

† F calcium from food sources, S calcium from supplements.
